# Sustainable and energy-efficient photocatalytic degradation of textile dye assisted by ecofriendly synthesized silver nanoparticles

**DOI:** 10.1038/s41598-023-29507-x

**Published:** 2023-02-09

**Authors:** Hemmat A. Elbadawy, Amel F. Elhusseiny, Seham M. Hussein, Wagih A. Sadik

**Affiliations:** 1 Chemistry Department, Faculty of Science, Alexandria University, 2 Bagdad Street, P.O. Box 2, Moharram Beck, Alexandria 21321 Egypt; 2 Materials Science Department, Institute of Graduate Studies and Research (IGSR), Alexandria University, Alexandria, Egypt

**Keywords:** Environmental sciences, Chemistry, Nanoscience and technology

## Abstract

In this study, we have touched on two goals of sustainable development, namely, the provision of clean water and sanitation and clean energy at acceptable prices, hoping for good health for all ages. A green economical method was used to prepare silver nanoparticles from chitosan biopolymer. AgNPs were fully characterized using UV–Vis, FTIR, XRD, HR-TEM, and EDX analysis. Different concentrations (0.02–0.18 g/L) of the nanoparticles were integrated into a mixture of heterogeneous nano photocatalysts TiO_2_ and ZnO (1:1 weight ratio) under UV irradiation for the photocatalytic degradation of Acid Red 37 textile dye to obtain clean water. The kinetic description of the performed photocatalytic process was presented assuming a pseudo-first-order reaction. The data revealed that increasing the concentration of AgNPs in the catalytic mixture showed a high apparent rate constant (k_app_) accompanied by an increase in the apparent quantum yield (%Q_app_), followed by dye destruction after a very short time (t_0.5_ = 3 min). Since the photocatalytic degradation process consumes electrical energy, the electrical energy per order (EE/O) was calculated, showing a low value of 20 kWh/m^3^/order, using 0.18 g/L AgNPs, indicating that the elicited photocatalytic degradation method is a sustainable one for the mineralization of the targeted dye.

## Introduction

Water pollution is a current concern worldwide, posing significant health and environmental hazards facing human lives. Water pollution by organic dyes or dye-based effluent is an imminent worldwide problem that causes a lack of clean and healthy water since most dyes are toxic with nonbiodegradable properties. Because dyes are used in many fields, such as the food, textile and leather industries, several processes, such as chemical precipitation, coagulation, flocculation, solvent extraction, biodegradation, membrane filtration, ion exchange, ozonation, electrochemical destruction, and adsorption, have been developed to reduce dye pollutants and protect the environment and aquatic life^1,2^. Unfortunately, these methods have high operating costs and are ineffective in accomplishing the total elimination of organic dyes from wastewater^3^.

In this direction, recent refined attention has been devoted to advanced oxidation processes (AOPs)^4–6^. Among these AOPs, heterogeneous photocatalysis is the most favorable procedure to breakdown organic pollutants into harmless compounds with virtual simplicity. The heterogeneous photocatalysis process depends on using a semiconductor catalyst activated by visible or ultraviolet radiation. When adsorption takes place, both hydroxyl radicals (HO^•^) and superoxide anion radicals (O_2_^·−^) are formed by the reaction of charge carriers (e^−^/h^+^ pairs) with H_2_O and O_2_^7^. These free radicals of high reactivity and high oxidation potential may react with nonbiodegradable organic toxic compounds, converting them into nontoxic products such as carbon dioxide and water or less toxic compounds^8^.

Moreover, photocatalytic degradation of organic dyes is considered an effective procedure using semiconductor oxides as catalysts under UV or visible irradiation^9–13^. Because of their excellent efficiency and relatively low cost, ZnO and TiO_2_ nanomaterials are the most often used semiconductors in photocatalytic processes^14–17^. Consequently, several works have shown that mixing ZnO and TiO_2_ causes a substantial improvement in photocatalytic activity^17–20^_._

Doping with noble metals such as silver or gold provides further improvement for the catalytic activities of ZnO or TiO_2_ photocatalysts. Thus, the rate of electron/hole recombination is slowed due to the presence of Schottky junctions, and their lifetime is lengthened as a result^21^. Furthermore, the Fermi level of titanium dioxide is higher than that of silver or gold; thus, when a noble metal is devoted to the surface of the catalyst, it acts as an electron scavenger. Hence, the oxygen photoreduction rate can be developed by these electrons and increase the quantity of photogenerated OH radicals^22^. Therefore, noble metal doping of semiconductor composites results in more operative photocatalysts^23^.

Silver nanomaterials are considered an excellent candidate among noble metals due to their rather low cost, great surface area to volume ratio, excessive electrical conductivity, optical and catalytic properties, nontoxicity, antimicrobial effect, and various applications in the field of health and the environment.

The presence of silver nanomaterials in contact with TiO_2_ and/or ZnO usually enhances the rate of the photodegradation process in an aqueous medium under ultraviolet irradiation^24–26^, which can be attributed to the synergism between TiO_2_/ZnO and silver loading heterojunctions^27–30^.

Recently, there has been a rise in interest in the "green" method of nanoparticle synthesis due to the fact that it is both cost-effective and environmentally friendly. The use of biopolymers in nanomaterial synthesis is gaining attention as a promising approach to achieving sustainability in the field of green nanotechnology^31–33^. The synthesis of silver nanoparticles via an eco-friendly technique is a vibrant role in nanotechnology studies. Nanoparticles preparation by several physicochemical approaches is extremely reactive and may cause great danger to the environment. Chitosan is a natural polysaccharide derived from chitin. Chitin is a biopolymer obtained from marine crustaceans, insects, and fungi shells. Great attention has been given to nanoparticles prepared from chitosan sources due to their remarkable biodegradability, significant biocompatibility and hydrophilic properties, which support their prospects for numerous applications^34^.

Herein, we report (i) the preparation and characterization of green silver nanoparticles (Ag NPs), (ii) the impact of green Ag NPs on the catalytic decomposition of the textile dye acid red 37 dye under ultraviolet irradiation, (iii) the influence of different amounts of green Ag NPs in the presence or absence of TiO_2_ and ZnO mixture under ultraviolet irradiation, and (iv) the calculation of the electrical energy per order and the quantum yield for all experiments. The photocatalytic activity is performed in a simple enclosed bench-scale batch photoreactor. The target textile dye, Acid Red 37 dye, was selected as a model pollutant, as it represents a common dye in the textile industry.

## Experimental section

### Materials

**Figure 1:**
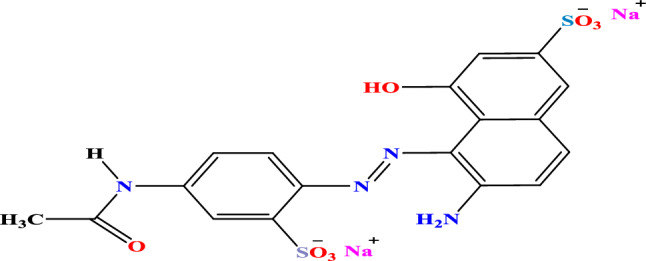
Molecular structure of Acid Red 37 dye.

The organic dye pollutant Acid Red 37, Fig. 1, was obtained from Chemajet. Zinc acetate was acquired from BDH. oxalic acid dihydrate (C_2_O_4_H_2_·2H_2_O) and 33% ammonia solution were procured from Adwic. The additional pure reagent 98% titanium tetrachloride was obtained from Yakuri/Osaka, JAPAN. Absolute ethanol (99.5%) was purchased from Scharlab. Glacial acetic acid was procured from Adwic, whereas silver nitrate was obtained from Fisher Scientific UK. Chitosan was purchased from Sigma Aldrich.

### Synthesis of ZnO and TiO_2_ NPs

Nanoparticles ZnO (24.4–27.8 nm) and TiO_2_ in its anatase form (7.62–17.42 nm) were prepared as reported in the literature^35^. 2.196 g, (0.01 mol) zinc acetate were dissolved in 60 ml ethanol and stirred at 60 °C for 30 min. 80 ml ethanolic solution of oxalic acid dihydrate (2.520 g, 0.02 mol) were stirred for 30 min at 50 °C and then added dropwise to a warmed solution of zinc acetate with continuous stirring for 1 h at the same temperature. A white gel was formed and dried at 80 °C. However, TiO_2_ was synthesized by the slow addition of 1.0 mL of TiCl_4_ to 10 mL of absolute ethanol in an ice bath (0–5 °C) with constant stirring for 30 min and allowed to cool at room temperature. Drops of (33%) ammonia solution were added until a white gel was obtained. The resulting sol–gel solution was washed with deionized water to remove any adhering chloride ions and then dried in an oven at 120 °C. ZnO and TiO_2_ were calcinated at 400 °C for 2 h under ambient atmosphere. The products were characterized in our previous work and reused in this study^35^.

### Synthesis of eco-friendly Ag NPs

**Figure 2:**
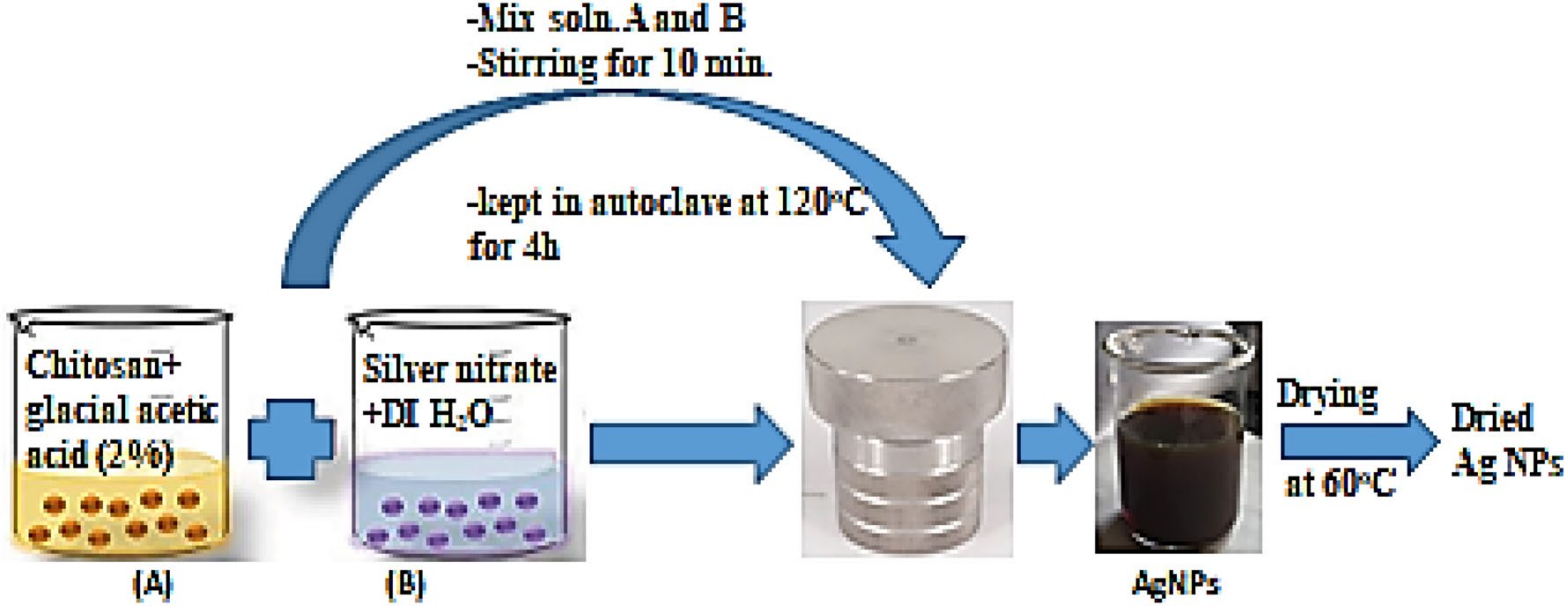
Representative diagram for the green preparation of Ag NPs.

Silver nitrate (500 mg) was dissolved in 100 mL deionized water, and 0.5% chitosan was prepared in 2% glacial acetic acid. Approximately 50 mL (0.5%) chitosan were stirred with 50 mL silver nitrate (0.5%) solution for 10 min. The achieved mixture was reserved in the autoclave at 120 °C for 4 h. The formation of the deep brownish-yellow solution indicated the formation of silver nanoparticles. To obtain dried nanoparticles, the solution was dried at 60 °C^36^. Figure 2 summarizes the synthesis procedure.

### Characterization of Ag nanoparticles

High-resolution transmission electron microscopy (HR-TEM) [A Japan-JEOL (Jem-2100)] was utilized to investigate the morphology and the mean particle size of the synthesized nanoparticles. The structure of the nanoparticles was confirmed by XRD analysis using XRD-Bruker D8 Advance E (Germany) working at room temperature with Cu (Kα) radiation of λ(1.5406 Å) spawned at 40 kV and 40 mA. The 2θ range was applied in the range of scan step size 3° ˂2θ˃ 80° with a scan rate of 4°/min at 25 °C. The elemental analyses of the prepared nanoparticles were attained using EDX (model: OXFORD_INCA PENTx3) and recorded with an electronic microscope (model: JEOL JSM 5410). The structural features of the prepared nanoparticles were studied by FTIR (Shimadzu-8400, Japan) as KBr discs in the extended wavenumber 400 to 4000 cm^−1^ with scan rate 2.0 cm^−1^ s^−1^.

### Photocatalytic degradation test

The reactor used for all photocatalytic experiments comprises a glass vessel containing a 0.1 L working volume of dye solution. The investigated dye was studied as an aqueous solution with a concentration of 1.0 × 10^–4^ M in distilled water as a wastewater pollutant model. The catalytic mixtures were prepared by simple mixing 0.5 g/L (1:1) (ZnO:TiO_2_) mixture with the appropriate weight of AgNPs (0.00, 0.02, 0.06, 0.12, and 0.18) g/L. The catalyst and dye solution were constantly stirred, and dry air at 3.5 L/min flow rate was fed into the reaction to aerate it. A tubular mercury vapor source of low-pressure with a 43 W total rating, total output ultraviolet at 13.4 W, 60 cm length and 254 nm, Volt arc Tubes Inc., USA) was utilized to irradiate the solution placed at 0.15 m from the source surface. The ultraviolet intensity was recorded at the central point of the solution, by a radiometer (Model UVX, UV Products Ltd, Cambridge) with a peak sensitivity sensor of 4.0 m W cm^−2^ at λ 254 nm. The dye solution samples were periodically taken from the vessel through a sample port, filtered via 0.2 µm polyether sulfone membrane, and then measured. The photodegradation tests were performed at a temperature of 22 ± 2 °C.

The decomposition of aqueous solutions of the studied pollutant (1.0 × 10^–4^ M) was performed by tracking the absorbance decrease of the maximum peak characteristic of Acid Red 37 dye at λ 504 nm using a UV‒visible spectrophotometer (Model T60 U, PG, UK). Both nanoparticles-containing samples were collected regularly from the photoreactor and measured after passing through a 0.2 µm polyether sulfone film. The degradation process was examined under ultraviolet light (254 nm) based on the assessment of the catalytic activity of (TiO_2_ and ZnO) mixture with different concentrations of silver nanoparticles (0.02 to 0.18 g/L).

The absorbance was calculated based on Beer–Lambert law^37^, The percentage degradation of the tested organic pollutant was calculated from Eq. (1):

1 $$\% {\text{ Degradation }} = \, [({\text{C}}_{{\text{o}}} - {\text{ C}})/{\text{C}}_{{\text{o}}} ] \times { 1}00 \, = \left[ {\left( {{\text{A}}_{{\text{o}}} {-}{\text{A}}} \right)/{\text{A}}_{{\text{o}}} } \right] \, \times { 1}00\% ,$$

where C_o_ and C are the dye concentrations before and after radiation time t, respectively. A_o_ and A are the values of absorbance of dye before and at time t of the reaction, respectively.

### Kinetics of degradation

The photodegradation kinetics of the investigated dye were evaluated by a Langmuir–Hinshelwood model^38^, Eq. (2), which was appropriate for the gaseous-solid interactions and liquid–solid interactions.

2 $${\text{ln}}\left( {{\text{C}}_{{\text{o}}} /{\text{ C}}} \right) \, = {\text{ k}}_{{{\text{app}}}} {\text{t,}}$$

where k_app_ indicates the apparent first-order rate constant, C and C_o_ are concentrations at time t and zero from the photocatalytic reaction, respectively. The apparent rate constant (k_app_) is estimated as the slope produced from the ln (C_o_/C) plot versus t. Subsequently, the half-life time (t_0.5_) of a pseudo-first-order reaction is deduced applying Eq. (3)

3 $${\text{t}}_{{0.{5}}} = \, 0.{693}/{\text{k}}_{{{\text{app}}}} .$$

The results of studying the influence of the catalyst concentration are determined with Eq. (4).

4 $${\text{k}}_{{{\text{app}}}} = {\text{K}}\left[ {{\text{catalyst}}} \right]^{{\text{n}}} ,$$

where (K) is the actual rate constant and (n) signifies the order of the reaction.

The following equation, Eq. (5), relates the initial reaction rate (R_initial_) to the apparent rate constant (k_app_) of a pseudo-first-order reaction:

5 $${\text{R}}_{{{\text{initial}}}} = {\text{ C}}_{{\text{o}}} {\text{k}}_{{{\text{app}}}} .$$

## Results and discussion

### Characterization of the synthesized green Ag nanoparticles

**Figure 3:**
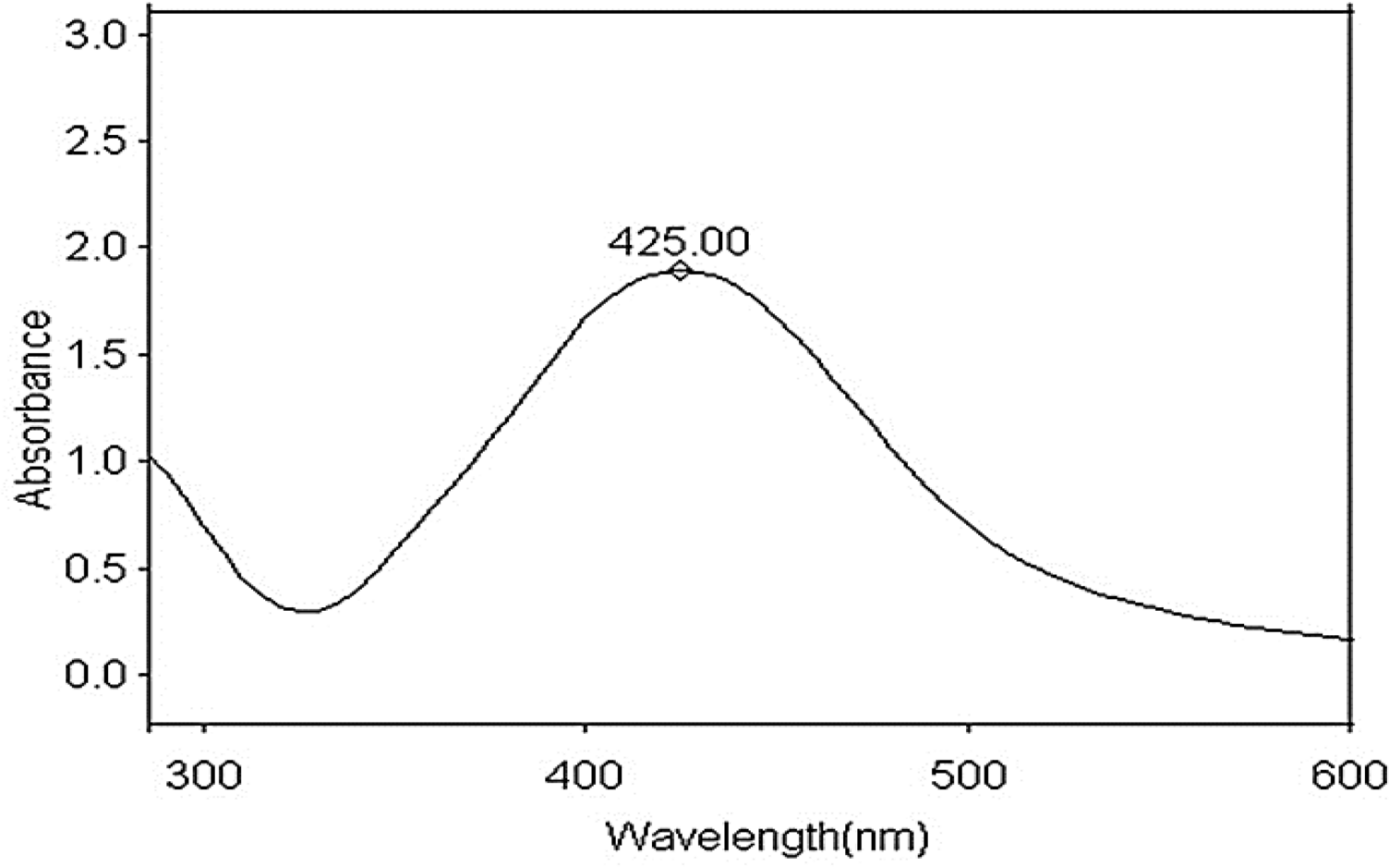
UV–visible absorption spectrum of silver nanoparticles stabilized in Chitosan.

The ultraviolet‒visible absorption spectrum of silver nanoparticles prepared from silver nitrate, reduced and stabilized by chitosan, is displayed in Fig. 3. The spectrum illustrated a strong absorption band centered at 425 nm owing to the surface plasmon resonance (SPR) of conducting electrons from the surface of AgNPs. No band was situated at approximately 335 and 560 nm, demonstrating the nonexistence of nanoparticles aggregation^36^.

**Figure 4:**
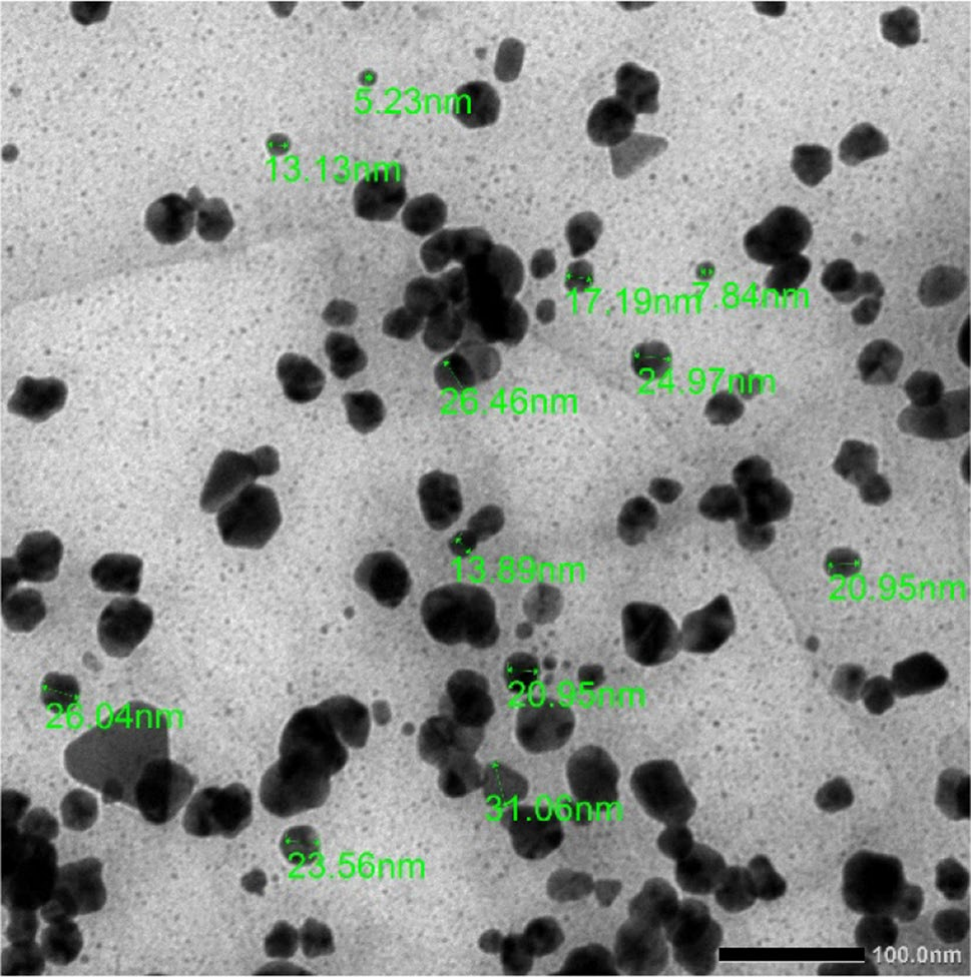
TEM image of AgNPs stabilized in Chitosan.

TEM images of AgNPs reduced/stabilized by chitosan are presented in Fig. 4. Frequently, silver nanoparticles have a spherical shape and are well distributed because of the chitosan matrix. The particle sizes ranged from 5 to 25 nm, and only a few particles were more than 30 nm.

**Figure 5:**
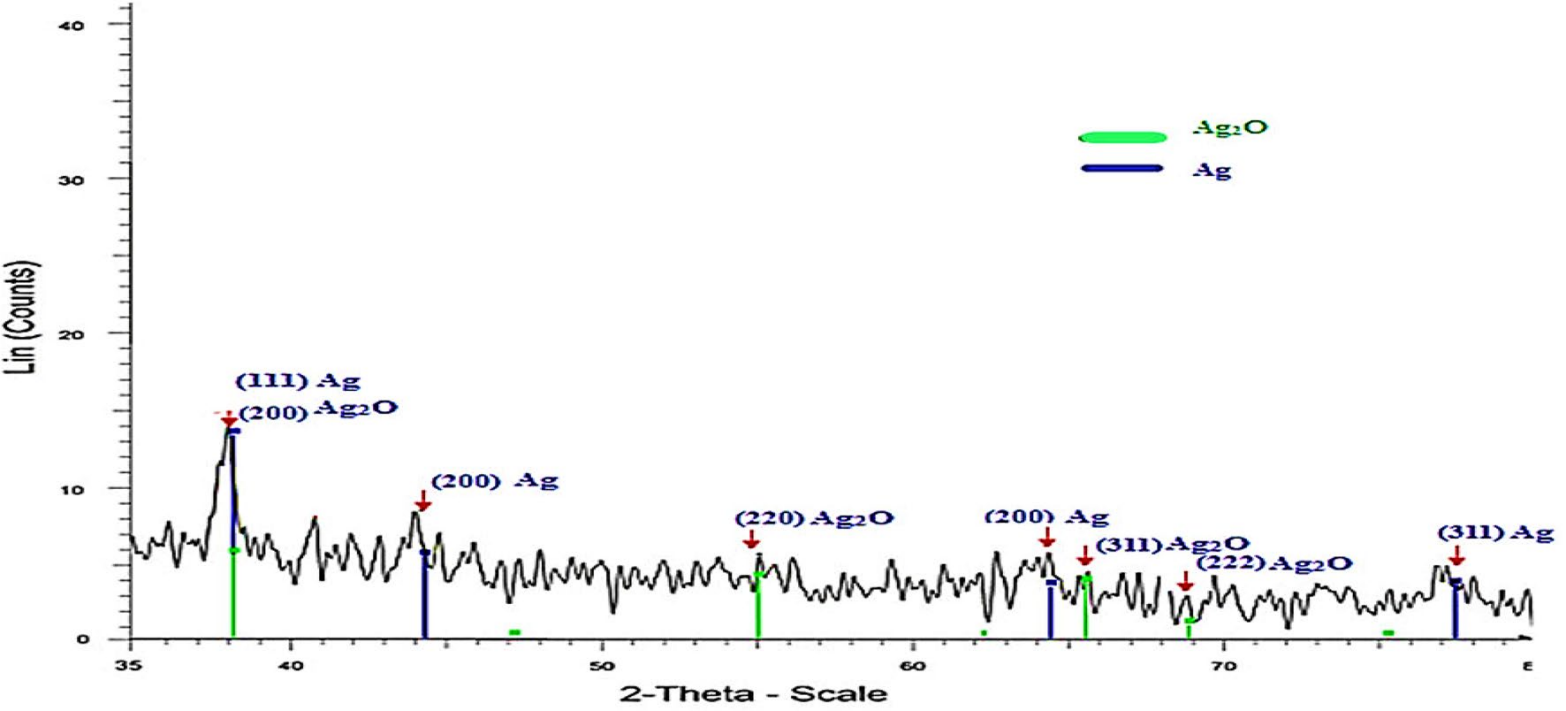
Typical XRD pattern of silver nanoparticles stabilized in Chitosan.

**Figure 6:**
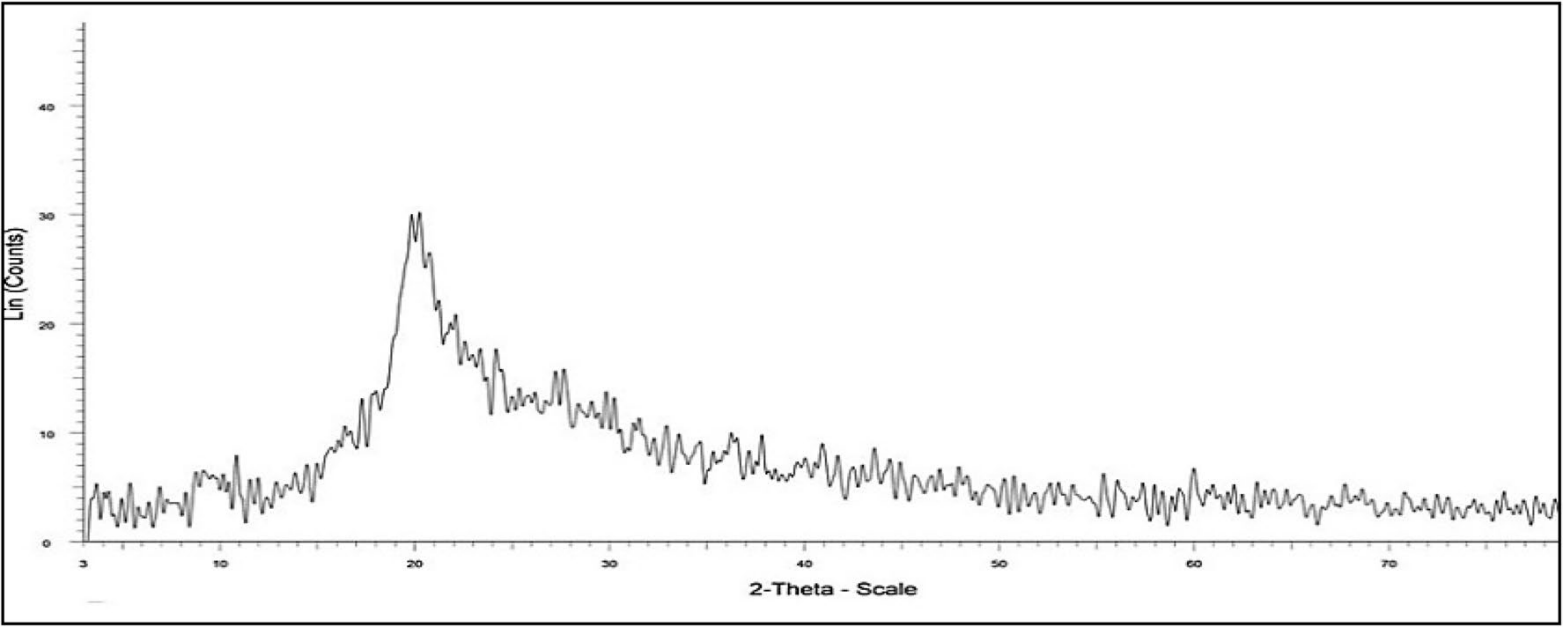
XRD pattern of chitosan.

The XRD spectrum of the synthesized AgNPs is displayed in Fig. 5. The XRD peaks at 38.1°, 44.3°, 64.7°, and 77.5° are related to the (111), (200), (200), and (311) crystal planes of cubic Ag(0), respectively (JCPDS 04*0783). Moreover, peaks that appeared at 38.1°, 54.9°, 65.4°, and 68.8° are perfectly due to the (200), (220), (311), and (222) crystal planes of cubic Ag_2_O, respectively (JCPDS 41-1104)^39,40^. The broadening of these peaks is mostly due to the presence of chitosan, which has an amorphous nature (Figs. 5, 6) and induces the loss of crystallinity of Ag NPs^41^. The XRD pattern indicated the existence of Ag and Ag_2_O. The formation of Ag_2_O may be related to the reaction of Ag(0) with oxygen from the environment^42^.

**Figure 7:**
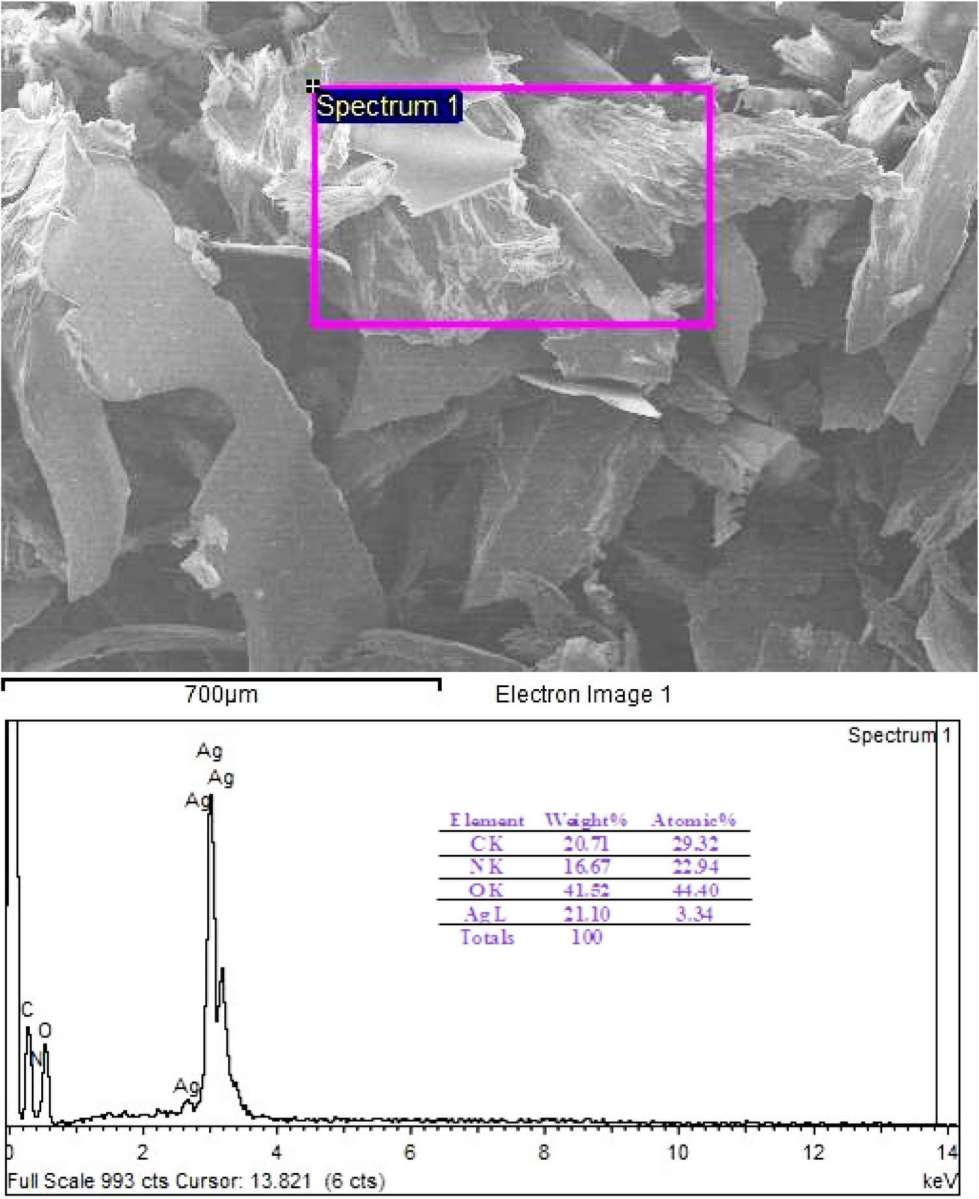
EDX of the prepared chitosan-based Ag NPs stabilized in Chitosan.

Figure 7 displays the EDX of the prepared nano-silver. The spectrum verified the presence of carbon, nitrogen, and oxygen, which are related to chitosan. The weight percentages of C, N, O, and Ag atoms are 20.71, 16.67, 41.52, and 21.0, respectively. No additional peaks are detected for other elements.

**Figure 8:**
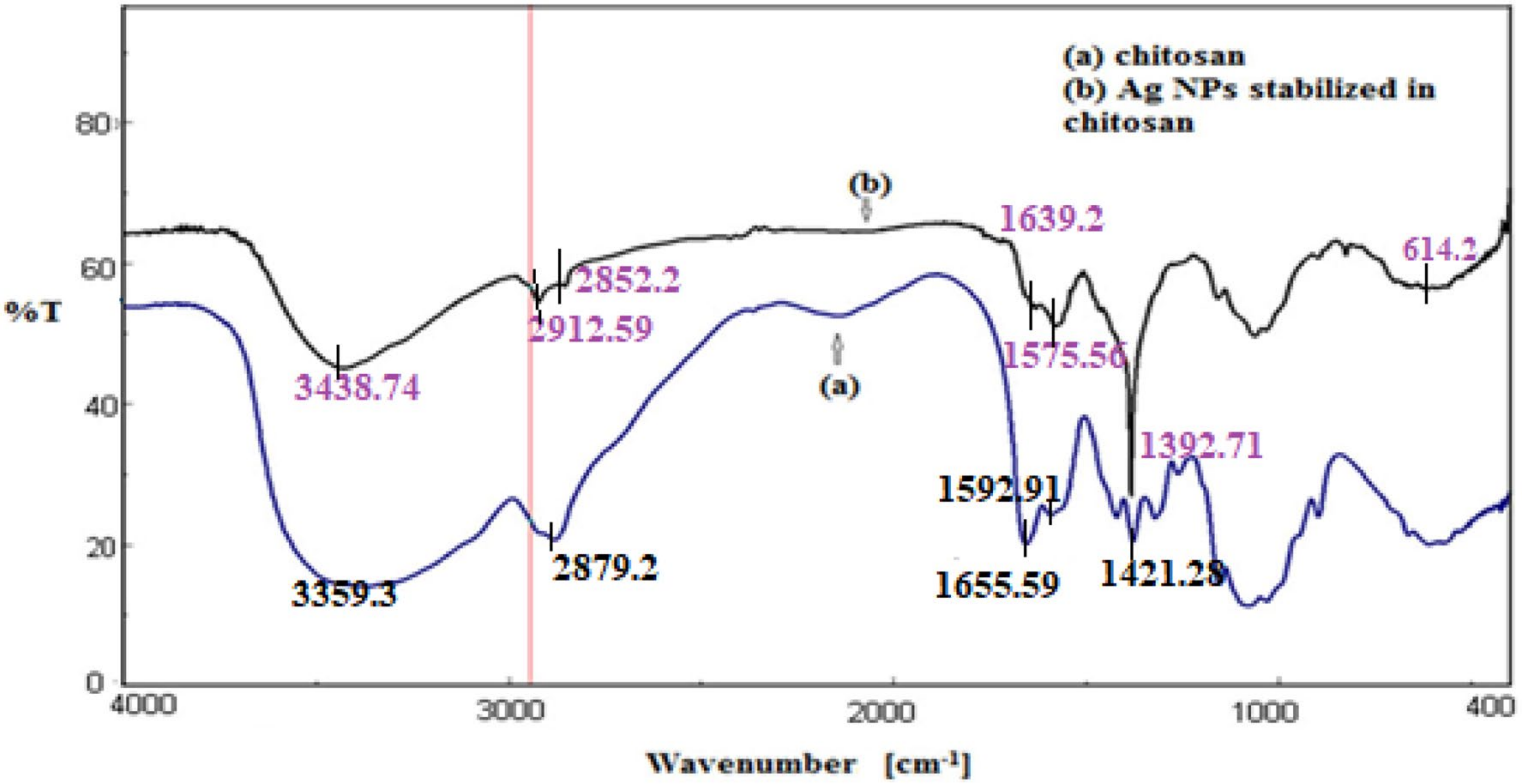
FTIR spectra of pure Chitosan and the prepared silver nanoparticles stabilized in Chitosan.

The FTIR spectra of pure chitosan and Ag NPs stabilized in chitosan are shown in Fig. 8a. The FTIR spectrum of chitosan showed characteristic bands at 3359, 2879, 1655, 1592, and 1421 cm^−1^ assigned to (–NH_2_, –OH), (–CH_2_, –CH_3_), (–CONH_2_), (NH_2_– bending vibration), and (–OH of primary alcohol), respectively. The broad band in the range of 3100–3500 cm^−1^ is ascribed to the overlap between the amino and –OH groups. The FTIR spectrum of AgNPs stabilized in chitosan is displayed in Fig. 8b. The bands at 1655 and 1592 cm^−1^, representing chitosan –CONH_2_ and –NH_2_ groups, vanished, and another weak band appeared at 1639 cm^−1^, signifying the physisorption linkage between silver and nitrogen atoms. The appearance of a broad band peak centered at 614 cm^−1^ may be due to the presence of Ag–O bonds^43^. The difference in both the peak position and the shape of the –OH and –NH_2_ groups at 3438 cm^−1^ confirms the reduction and stabilization process^36^.

### Photocatalytic degradation activity

#### Effect of Ag nanoparticles

**Figure 9:**
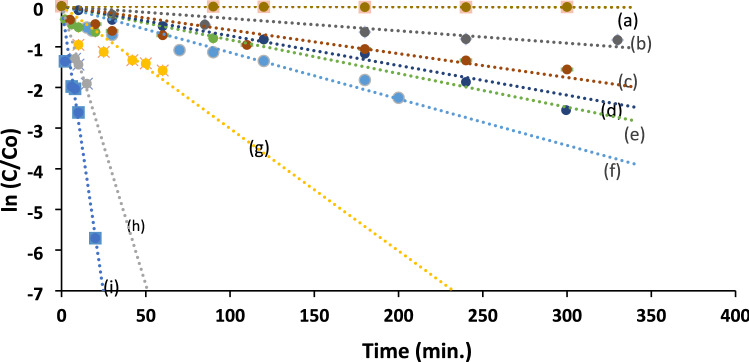
Photocatalytic degradation of 1.0 × 10^–4^ M textile dye, under the influence of (**a**) UV irradiation only, (**b**) UV/0.5 g ZnO, (**c**) UV/0.5 g TiO_2_, (**d**) UV/0.5 g (ZnO–TiO_2_), (**e**) UV/Ag (0.02 g/L), (**f**) UV/0.5 g (ZnO–TiO_2_)/(0.02 g/L) AgNPs, (**g**) UV/0.5 g (ZnO–TiO_2_)/(0.06 g/L)AgNPs, (**h**) UV/0.5 g(ZnO–TiO_2_)/(0.12 g/L)AgNPs, and (**i**) UV/0.5 g(ZnO:TiO_2_)/(0.18 g/L)AgNPs.

The impact of green synthesized Ag NPs on the photocatalytic decomposition of aqueous Acid Red 37 dye was examined under ultraviolet irradiation. The AOP study included using Ag Nps in the absence and presence of a mixture of TiO_2_–ZnO (1:1) and their photocatalytic performance were compared with other systems, such as UV/TiO_2_, UV/ZnO and UV/(TiO_2_–ZnO) (1:1) Fig. 9. All experiments were performed in a batch photoreactor.

**Figure 10:**
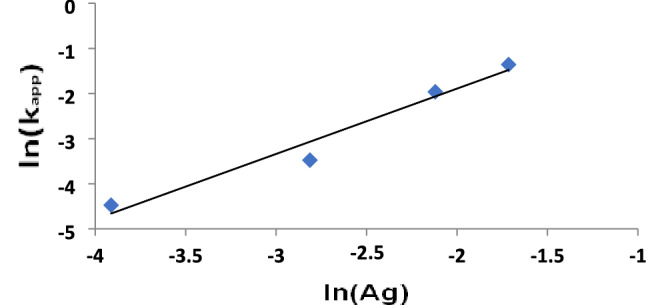
Plot of ln k_app_ (min^−1^) versus ln [Ag] for photocatalytic degradation of the textile dye.

The photocatalytic degradation process was found to be more operative in (UV/Ag NPs) and (UV/(TiO_2_–ZnO & x Ag NPs) systems, where x = different amounts of green AgNPs added from 0.02–0.18 g/L. The effectiveness of UV/Ag NPs system may be related to their low recombination rate^44^. The addition of Ag NPs, even with low concentration, 0.02 g/L to (TiO_2_–ZnO) (1:1), using UV irradiation, enhanced the degradation rate of the dye compared to the other examined systems. Moreover, increasing the concentration of Ag NPs added to UV/(TiO_2_–ZnO) mixture from 0.02 to 0.18 g/L led to dye destruction after a very short time (t_0.5_ = 3 min using 0.18 g/L). The reaction rate order for Ag NPs was 1.4 (Fig. 10, Table 1).

**Table 1:** Kinetic parameters for the photocatalytic degradation of 1.0 × 10^–4^ M acid red 37 dye, upon UV irradiation.

Catalyst	Concentration	k_app_ (min^−1^)	T_0.5_ (min)	R_initial_ = C_o_k_app_ (mol L^−1^ min^−1^)	Apparent reaction order
	(g/L)				
–	**–**	1.0 × 10^–4^	6930	9.7 × 10^–9^	
TiO_2_	0.5	5.9 × 10^–3^	117	5.9 × 10^–7^	
ZnO	0.5	3.0 × 10^–3^	231	3.1 × 10^–7^	
TiO_2_–ZnO (1:1)	0.5	7.3 × 10^–3^	95	7.3 × 10^–7^	
Ag NPs	0.02	8.3 × 10^–3^	83.5	8.1 × 10^–7^	–
(TiO_2_–ZnO and x Ag NPs)	x = 0.02	1.1 × 10^–2^	61	1.1 × 10^–6^	1.4
	x = 0.06	3.1 × 10^–2^	22	3.1 × 10^–6^	
	x = 0.12	14.1 × 10^–2^	5	1.4 × 10^–5^	
	x = 0.18	26.0 × 10^–2^	3	2.5 × 10^–4^	

#### Mechanism of photocatalytic degradation

**Figure 11:**
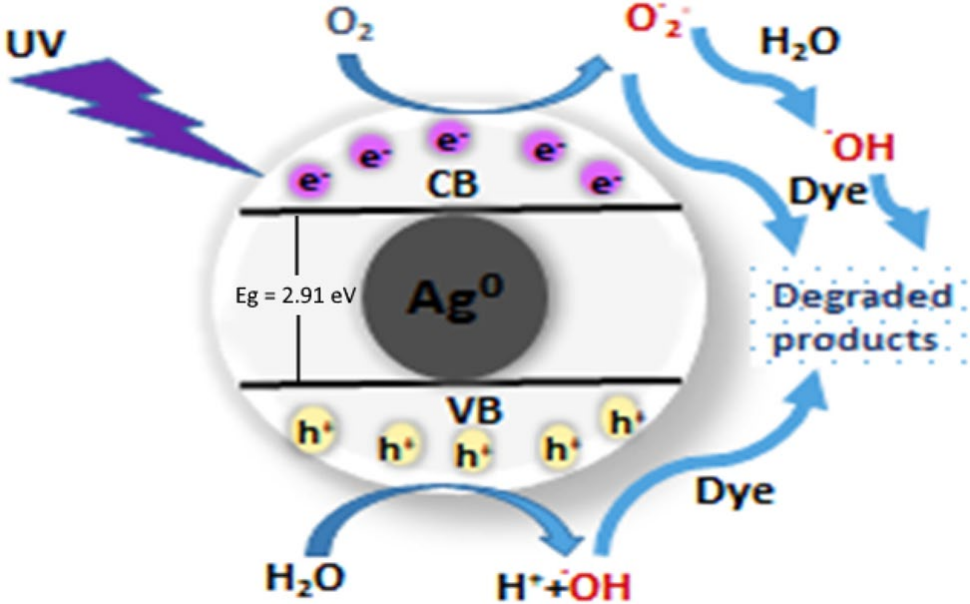
Proposed mechanism of Ag NPs-role as a photocatalyst, upon UV-irradiation.

**Figure 12:**
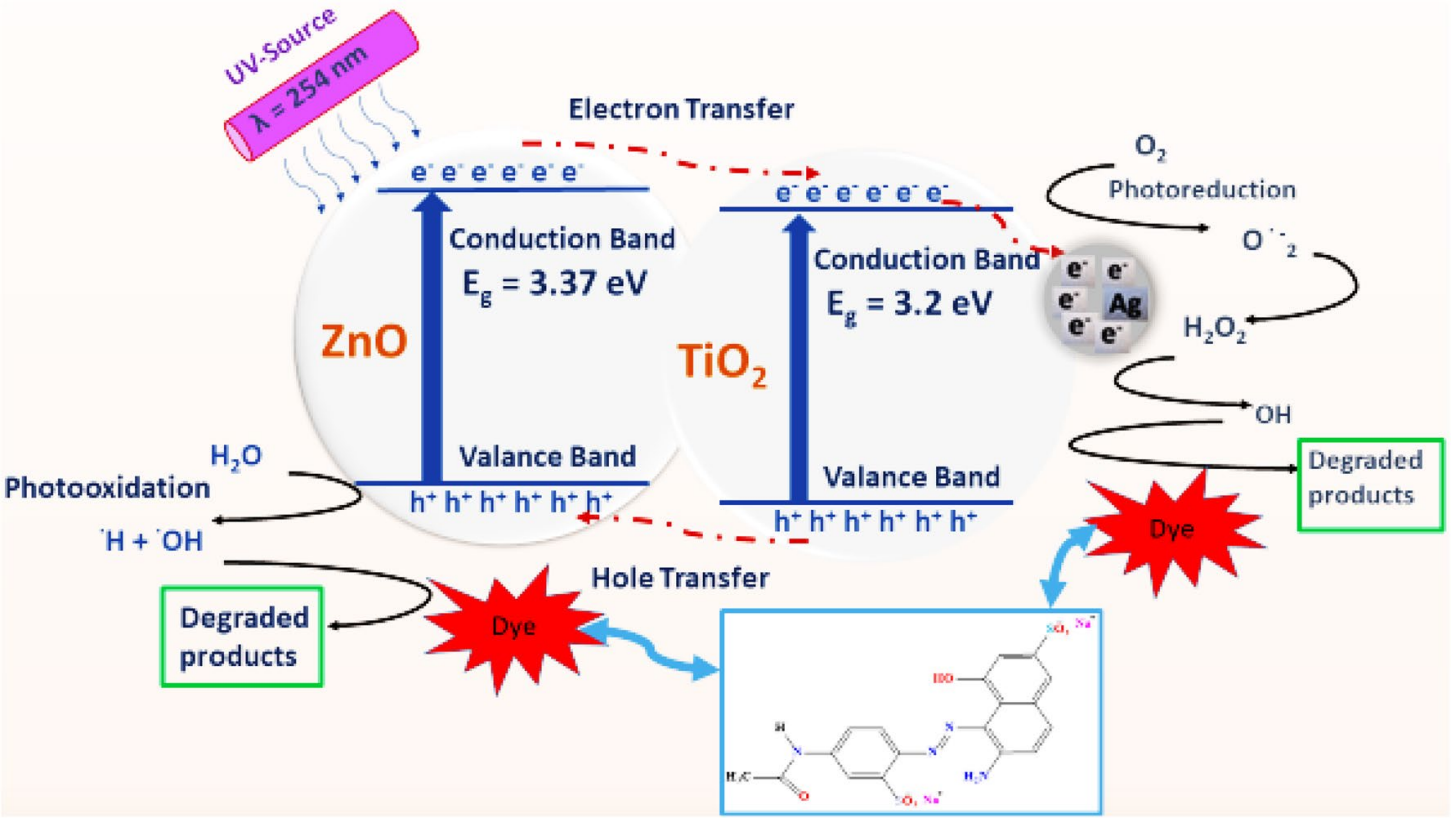
Proposed mechanism of the investigated dye degradation using UV/(TiO_2_–ZnO & x Ag NPs) system.

A proposed mechanism of the degradation method of the investigated dye using the UV/Ag NP system is presented in Fig. 11. The energy of the ultraviolet radiation over the surface of the nanoparticles causes electrons excitation from the valence band (VB) up to the conduction band (CB), leaving positive holes (h^+^) instead. The photogenerated species (h^+^/e^−^) interact with water from the medium, producing extremely reactive radicals (^**·**^OH and O^**·**^_2_^−^) that result in dye degradation^45^. The photocatalytic consequence of the (TiO_2_–ZnO and x Ag NPs) system can be explained as demonstrated in Fig. 12. Upon irradiating the system with ultraviolet photons, electrons in the valence band of zinc oxide are generated and photoexcited to the conduction band (CB), leaving the same amounts of holes in the valence band (VB). Similarly, when TiO_2_ is exposed to UV light, the production of photoexcited electrons and holes occurs. Because the conduction band energy of zinc oxide is greater than that of titanium dioxide and the conduction band energy of titanium dioxide is greater than silver Fermi level, photoinduced electrons move from zinc oxide to titanium dioxide and then from titanium dioxide to Ag NPs. At the same time, holes can transfer from the titanium dioxide valence band to that of zinc oxide. To reveal the role of Ag NPs catalytic effect, we can follow the impact of UV irradiation in the absence of Ag NPs. The holes in VB either directly participate in the breakdown or react with hydroxyl groups to yield (**·**OH). Correspondingly, the electrons can reduce oxygen-forming superoxide ions (O_2_^**·**−^), which can produce hydrogen peroxide (H_2_O_2_) from the aqueous solution and hydroxyl radicals (**·**OH), improving photocatalytic dye degradation. Therefore, the combination of titanium dioxide and zinc oxide reduces charge recombination, resulting in a longer lifetime of photogenerated electron–hole pairs. The addition of Ag NPs, so that the catalytic system can be presented as (TiO_2_–ZnO and x Ag NPs), gives an extra effect, where Ag NPs act as an electron scavenging agent that reduces the recombination rate of photoinduced electrons and holes due to the production of a Schottky barrier at Ag-semiconductor interface. Therefore, the increase in electron density on silver reduces oxygen to form superoxide radicals. Furthermore, this enhances charge separation, and more photogenerated holes could be included in the photocatalytic degradation process^46–49^. The existence of silver oxides, investigated from the XRD spectrum, also enhances photocatalytic activity^48,50^.

Hence, TiO_2_, ZnO, and Ag NPs synergistic effect may bring the hopeful applicant to environmental applications, particularly wastewater treatment.

The following equations summarize the proposed mechanisms for (A) AgNPs alone and (B) in presence of /(TiO_2_/ZnO) mixture, under UV radiation:

**Table:** 

(A) UV/AgNPs System	(B) UV/(TiO_2_/ZnO)/AgNPs system
$Ag \stackrel{UV, 254nm }{\to } {e}^{-}\left(CB\right)+{h}^{+}\left(VB\right)$	$ZnO \stackrel{UV, 254nm }{\to } {e}^{-}\left(CB\right)+{h}^{+}\left(VB\right)$
${O}_{2}+{e}^{-}\to {O}_{2}^{-.}$	$Ti{O}_{2} \stackrel{UV, 254nm }{\to } {e}^{-}\left(CB\right)+{h}^{+}\left(VB\right)$
${O}_{2}^{-.}+{H}_{2}O \to \dot{OH}$	${e}^{-}\left(CB\,ZnO\right) \stackrel{transfer\,to}{\to } \left(CB\,Ti{O}_{2}\right)$
$\dot{OH}+dye\to Degradation$	${h}^{+}\left(VB\,Ti{O}_{2}\right) \stackrel{transfer\,to}{\to } \left(VB\,ZnO\right)$
${h}^{+}\left(VB\right)+{H}_{2}O \to \dot{O}H$	${e}^{-}\left(CB\,Ti{O}_{2}\right) \stackrel{transfer\,to}{\to } AgNPs\,({e}^{-})$
$\dot{O}H+dye\to Degradation$	$AgNPs\,\left({e}^{-}\right)+ {O}_{2}+{e}^{-}\to {O}_{2}^{-.}$
	$${H}_{2}{O}_{2}\stackrel{ UV }{\to } 2\,\dot{O}H$$
	$$\dot{O}H+dye\to Degradation$$

### Photocatalytic reaction efficiency

The efficacy of the degradation method is often measured by quantum yield (Q), which can be related to the product formation or reactant disappearance rate compared to the number of absorbed photons per unit of time. The photocatalyst is known to be the absorbing light species that can cover a solid support in contact with the reactants or spread as a slurry in an aqueous medium. A significant portion of incident photons is scattered or reflected by using coated or dispersed catalysts. It is difficult to explore the quantum yields in heterogeneous photocatalysis, making experimental detection of the quantity of light absorbed by the photocatalyst impossible.

Accordingly, the apparent quantum yield (Q_app_) is frequently described as an alternative parameter and well-defined as Eq. (6)^51,52^:

6 $$\begin{array}{c}Apparent \; quantum \; yield\\ \left({Q}_{app}\frac{mol}{Einstein}\right)\end{array}=\frac{Rate \; of \; disappearence \; of \; reactant \; molecules}{Rate \; of \; incident \; photons \; inside \; reactor \; cell}=\frac{{k}_{app}{C}_{o}}{I},$$

where C_o_ is the initial concentration of the dye, I is the total intensity of incident photons entering the reactor cell, and k_app_ is the apparent first-order rate constant.

Table 2 shows the % apparent quantum yield (%Q_app_ = *100 Q*_*app*_*)* for the examined procedures. The achieved data show that Q_app_ for the TiO_2_–ZnO & x Ag NPs system is higher than that for the other studied photocatalytic systems. Its values increase with increasing concentration of Ag NPs added to the TiO_2_–ZnO mixture. This could be attributed to the developed electronic trapping by the added Ag NPs, leading to a higher photocatalytic degradation.

**Table 2:** The apparent rate constants, electrical energy per order, and the apparent quantum yield for the photocatalytic degradation of 1.0 × 10^–4^ M dye pollutant upon UV irradiation.

Catalyst	Concentration (g L^−1^)	K_app_ (min^−1^)	EE/O (kWh/m^3^)	%Q_app_
–	–	1.0 × 10^–4^	50,000	7.6 × 10^–5^
TiO_2_	0.5	5.9 × 10^–3^	714.2	0.47
ZnO	0.5	3.0 × 10^–3^	1666.6	0.24
(TiO_2_–ZnO) (1:1)	0.25 + 0.25	7.3 × 10^–3^	714.29	0.58
Ag NPs	0.02	8.3 × 10^–3^	625.0	0.64
(TiO_2_–ZnO & x Ag NPs)	x = 0.02	1.1 × 10^–2^	384.6	0.87
	x = 0.06	3.1 × 10^–2^	166.7	2.62
	x = 0.12	14.1 × 10^–2^	36.63	11.0
	x = 0.18	26.0 × 10^–2^	20.00	20.2

### Electrical energy evaluation

**Figure 13:**
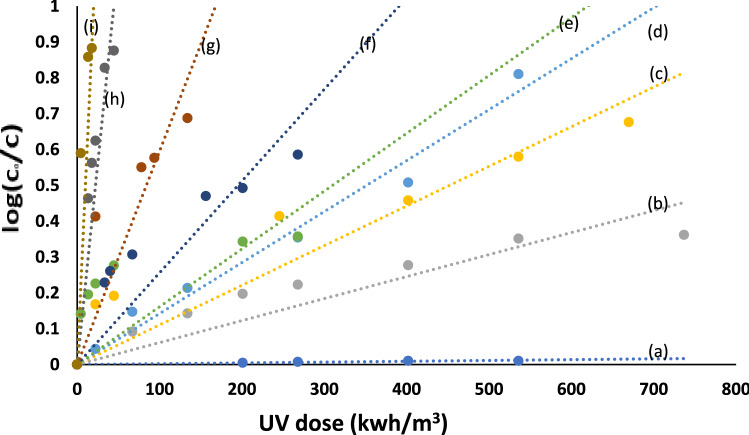
Plot of log (C_o_/C) versus UV dose (kwh/m^3^) for degradation of (1.0 × 10^-4^ mol/ L) Acid Red 37 dye in: (**a**) absence of catalysts, (**b**) presence of ZnO, (**c**) presence of TiO_2_, (**d**) presence of ZnO–TiO_2_ mixture, (**e**) presence of 0.02 g/L chitosan-based Ag NPs, (**f**) (ZnO–TiO_2_) and 0.02 g/L Ag NPs, (**g**) (ZnO–TiO_2_) and 0.06 g/L AgNPs, (**h**) (ZnO–TiO_2_) and 0.12 g/L Ag NPs, (**i**) (ZnO–TiO_2_) and 0.18 g/L AgNPs.

Several important parameters, such as electrical energy consumption, effluent quality, economics, and cost, are considered to have a dynamic role in the assortment of suitable waste treatment technologies. Thus, photocatalytic degradation of organic pollutants is connected to electrical energy, which acts as the main component of the operating expenses^53^. Consequently, the quantity of electrical energy (kWh) needed to decrease the concentration of the textile by 90% by one order of magnitude in 1 m^3^ of polluted water, known as electrical energy per order, EE/O, is a significant parameter in this direction. The EE/O value permits a fast estimation of the electrical energy expenses and confirms the essential total power. It is calculated from the plotting of log (C_o_/C) versus the amount of ultraviolet light (Fig. 13). The ultraviolet doses were estimated for applied AOPs using Eq. (7), considering pseudo first-order degradation kinetics^52^. Accordingly, EE/O can also be determined from ultraviolet dose using Eq. (8) ^54^.

7 $$UV \; dose=\frac{lamp \; power \left(kW\right) \times time\left(h\right) \times 1000}{Treated \; volume\left(L\right)},$$

8 $${\text{EE}}/{\text{O }} = {\text{ UV}}\,{\text{dose}}/{\text{log }}\left( {{\text{C}}_{{\text{o}}} /{\text{C}}} \right).$$

For all the investigated systems, the EE/O values are compiled in Table 2. The addition of Ag NPs to 0.5 g of 1:1 (TiO_2_–ZnO) mixture under ultraviolet irradiation showed a lower EE/O value relative to other applied systems. Increasing the concentration of Ag NPs decreased the EE/O value even more. The calculated EE/O value for electric energy was 20 kWh/m^3^/order using 0.18 g/L Ag NPs when the initial concentration of the Acid Red 37 dye was 1.0 × 10^−4^ M, and compared with electric consumption, the oxidant cost was insignificant.

## Conclusions

The acid red 37 dye was subjected to catalytic degradation upon UV radiation, applying the advanced oxidation process (AOP). TiO_2_, ZnO, and chitosan-based Ag nanoparticles were prepared, fully characterized, and used for the photocatalytic degradation of the dye. The study included UV/(TiO_2_), UV/(ZnO), UV/(TiO_2_–ZnO), UV/Ag NPs and (TiO_2_–ZnO and x Ag NPs) systems. The most effective system was TiO_2_–ZnO and x Ag NPs, and increasing the concentration of Ag NPs from x = 0.02 to x = 0.18 g/L enhanced the dye destruction with t_0.5_ (61.0–3.0 min). Subsequently, the combination of TiO_2_ and ZnO decreases the tendency of charge recombination, resulting in photogenerated electron–hole pairs with an extended lifetime. Moreover, Ag NPs act as electron trapping agent that improves hydroxyl radical formation and the speed of reactions. The presence of silver oxide also contributes to the development of photocatalytic action. The synergistic influence of ZnO, TiO_2,_ and silver NPs may bring about expectant candidates for environmental applications, especially in wastewater treatment. The efficiency parameters apparent quantum yield (Q_app_) and electrical energy per order (EE/O) were assessed, and their values were related for the studied systems. The data proved that (TiO_2_–ZnO and x Ag NPs) system provides the highest %Q_app_ that ranges from (0.870–20.2), with the lowest values of EE/O (384.6–20.00) with increasing concentration of Ag NPs added (0.02–0.18) g/L.

## Data Availability

The datasets used and/or analyzed during the current study are available from the corresponding author on reasonable request.
